# Aortic valve-sparing procedure—a durable choice in liver transplantation recipient

**DOI:** 10.1007/s11748-020-01434-y

**Published:** 2020-07-12

**Authors:** Andrea De Martino, Stefano Pratali, Paola Carrai, Stefania Petruccelli, Paolo De Simone, Uberto Bortolotti

**Affiliations:** 1 Sezione Autonoma Di Cardiochirurgia Universitaria, Section of Cardiac Surgery, Pisa University Hospital, Via Paradisa, 2, 56124 Pisa, Italy; 2 Liver Transplant Surgery Unit, Pisa University Hospital, Pisa, Italy

**Keywords:** David procedure, Liver transplantation, Anticoagulation

## Abstract

A 44-year old man with aortic regurgitation and aneurysm of the ascending aorta underwent an aortic valve-sparing procedure as a durable treatment before liver transplantation. Since patients with chronic liver failure are at high risk of hemorrhagic complications at time of major surgery, while management of warfarin administration may still represent a concern, the choice of a cardiac procedure which avoids any anticoagulant treatment appeared justified.

## Introduction

Valve-sparing procedures are currently the treatment of choice for patients with aortic regurgitation and aneurysm of root and ascending aorta, since repairing the native aortic valve combines the advantages of long-term valve durability with avoidance of anticoagulant treatment [1]. Furthermore, should a major extracardiac procedure be required during follow-up, absence of anticoagulation would minimize any potential hemorrhagic risk, especially for patients requiring solid organ transplantation such as the liver. While various open heart procedures have been reported after or during liver transplantation (LT) [2, 3], little is known about patients undergoing LT after major cardiac surgery. We report here a patient who underwent LT and re-transplantation after a valve sparing operation, underlining the advantage of this procedure in this setting.

## Case report

**Fig. 1:**
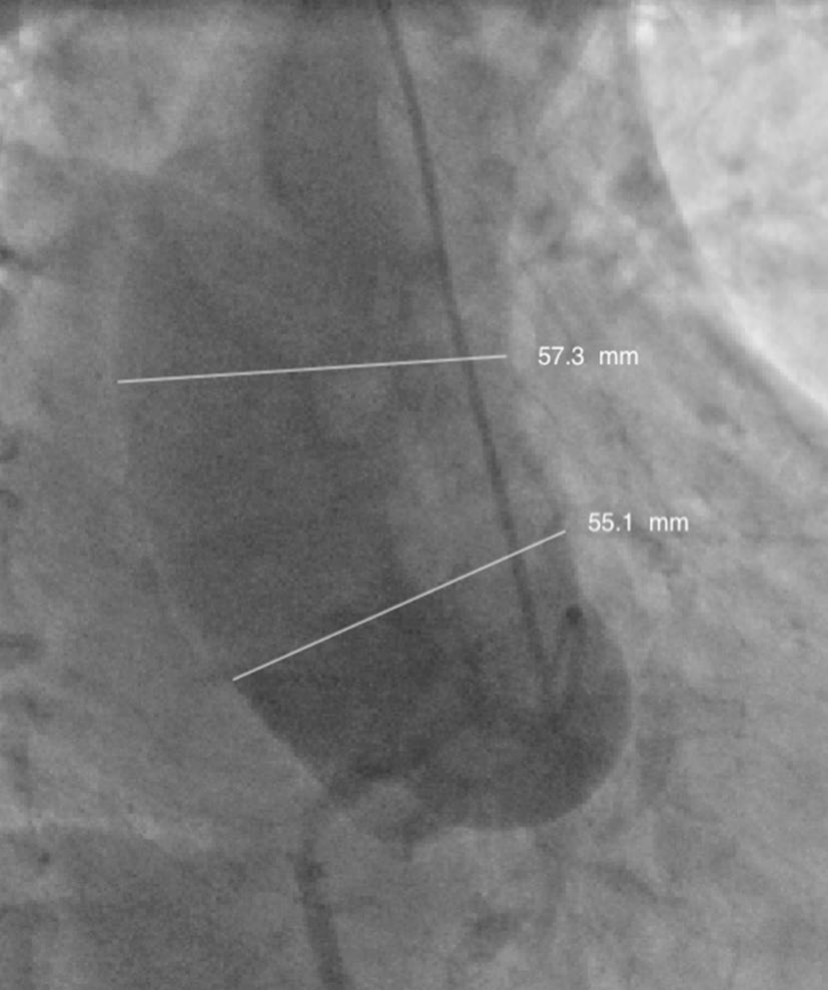
Preoperative angiography showing a dilated ascending aorta (57 mm) and root (55 mm at the sino-tubular junction)

A 44-year old man affected by liver cirrhosis related to hepatitis B virus (HBV) found to have an aneurysm of the ascending aorta and root during work-up for LT. A transthoracic 2D echo also showed moderate aortic regurgitation and a moderately depressed left ventricular function (ejection fraction 45%, end-diastolic diameter 56 mm). An angio-computed tomography confirmed the presence of an aortic aneurysm, while angiography showed an enlarged aorta with 57 mm in the largest tract of the ascending aorta and 55 mm at the sino-tubular junction, 50 mm at the root, 36 mm at ventriculo-aortic junction (Fig. 1), and presence of right coronary artery stenosis. His blood tests showed marked thrombocytopenia (45,000/µL), prolonged partial prothrombin time, and reduced prothrombin activity (77%), AST 73 U/L, ALT 83 U/L, bilirubin 0.9 mg/dL. Based on his cardiovascular status, the patient was referred to cardiac surgery. Surgical repair was achieved through median sternotomy with moderately hypothermic cardiopulmonary bypass. A valve-sparing procedure was performed according to the technique described by El Khoury et al. [4], using a 28 mm Valsalva graft (Vascutek Ltd., Inchinnan, Scotland), with reimplantation of the aortic valve inside the graft and reattachment of the coronary buttons, while the right coronary artery was grafted with a segment of saphenous vein. Distal aortic anastomosis was performed with a continuous suture reinforced with a Teflon felt strip with aortic cross-clamp. Before release of the aortic clamp all sutures were generously covered with fibrin sealant (Tisseel™, Baxter International Inc., IL, USA). CPB time was 133 min and aortic cross-clamp time 121. Total blood loss was 900 mL with need of 500 mL of red blood cells transfusion; blood tests revealed thrombocytopenia (nadir 35,000/µL on 3rd postoperative day), reduced prothrombin activity (62%), bilirubin 2.3 mg/dL, AST 1242 U/L, ALT 1379 U/L (peak on 1st postoperative day). The postoperative course was substantially uneventful and prior to discharge a 2D transthoracic echo showed no evidence of aortic regurgitation and good cusp coaptation. No anticoagulants or antiplatelet drugs were administered. At 6-months follow-up, an initial recovery of ejection fraction (50%) and decrease of left ventricular diameter (51 mm) were observed. 8 months thereafter the patient was listed for deceased donor LT. Whole-size LT was performed nine months after cardiac surgery from a 74-yearold brain dead donor. Surgery was carried out with vena cava replacement and veno-venous by-pass while the biliary anastomosis was performed with a T-tube. Total postoperative blood loss was 800 mL. The initial post-operative course was uneventful and the patient received induction immunosuppression with anti-CD25 monoclonal antibodies (basiliximab, Simulect™, Novartis, Origgio, Italy), coupled with steroids, mycophenolate mofetil and tacrolimus, while anti-HBV prophylaxis included entecavir and anti-HBs immunoglobulin. Seven days post-transplantation the patient, despite low-molecular-weight-heparin prophylaxis, was diagnosed with thrombosis of the hepatic artery and re-listed for urgent transplantation. One day after the patient underwent liver re-transplantation form a 55-year old brain-dead donor. Post-LT drainage was 900 mL and the post-operative course uneventful. Nine years after transplantation, the patient is alive with no evidence of recurrent HBV disease and normal aortic valve function.

## Discussion

Patients with end-stage liver disease undergoing heart surgery are reported with successful early outcome but poor late survival due to persistent liver dysfunction [5]. On the other hand, patients requiring LT and affected by cardiac disease with clear cut surgical indications are still a matter of ongoing debate. Due to improvements in surgical techniques and postoperative management, the number of patients with end-stage liver disease referred for LT is steadily increasing. Chronic cardiac dysfunction is common in cirrhotic patients [2], and since LT induces severe stress to the cardiovascular system, a normal cardiac function is of paramount importance for subjects undergoing this procedure [2]. Various types of cardiac operations have been performed after or simultaneously with LT [2, 3, 5]. However, open heart procedures prior to LT have been reported only in patients undergoing myocardial revascularization [6].

There are 2 reports in the literature on patients undergoing transcatheter procedures before LT. Coverstone et al. [7] described 4 patients who underwent aortic balloon valvuloplasty for severe aortic stenosis pre-LT, while Cabasa et al. [8] reported transcatheter aortic valve implantation (TAVI) in another patient with aortic regurgitation listed for LT (Table 1). To the best of our knowledge, our patient is the first to undergo an open surgical procedure involving the ascending aorta and the aortic valve prior to LT. We also considered various possible alternative procedures, all of which were discarded. A TAVI was obviously considered contraindicated by the presence of an aortic aneurysm, but nevertheless it would have never been selected due to the young age of the patient and the still unknown durability of biological devices used for TAVI [9, 10]; a modified Bentall procedure (MBP) with a mechanical conduit would have exposed the patient to the unjustified risks of life-long anticoagulation; finally, a MBP with a biological prosthesis would have avoided chronic anticoagulants, providing a procedure-related durability not superior to that of a valve-sparing procedure, despite an expected excellent survival after LT for HBV [11]. Indeed, our experience with aortic valve-sparing procedure has shown a 98% freedom from procedure-related reoperations at 10 years [1], while others have observed 95% freedom from reoperations on the aortic valve at 18 years [12]. Therefore, based on the previous considerations, in our patient, a valve-sparing procedure was considered the best option, combining the advantages of long-term durability and avoidance of life-long anticoagulation, which is generally contraindicated in patients with liver failure [13]. But, after all, why replace an anatomically normal aortic valve in a young patient with aortic regurgitation and dilated aortic root and ascending aorta?

**Table 1:** Summary of patients undergoing open surgery or endovascular treatment for valvular disease before liver transplantation

Author	Year	Age (years), sex	Disease	Procedure	Outcome	Interval to LT	Follow-up
Coverstone et al. [7]	2014	58,F	AS, cirrhosis	ABV	Alive	38 h	SAVR
		63, M	AS, hemochromatosis	ABV	Alive	4 months	SAVR
		56, M	AS, liver steatosis	ABV	Alive	6 h	SAVR
		58, M	AS, hepatitis C	ABV	Alive	20 h	Dead
Cabasa et al. [8]	2016	64, M	AR, hepatic carcinoma	TAVI	Alive	2 months	Alive, 2 months
Present case	2020	44, M	AR, cirrhosis	Aortic valve- sparing	Alive	9 months	Re-LT on POD 8 Alive at 9 years

*M* male, *F* female, *AS* aortic stenosis, *AR* aortic regurgitation, *ABV* aortic balloon valvuloplasty, *SAVR* surgical aortic valve replacement, *TAVI* transcatheter aortic valve implantation, *LT* liver transplantation

Management of coagulation and anticoagulation in LT candidates has been discussed in a recent review article [13]. Despite the care of patients with chronic liver disease significantly evolving in recent years, impairment in hemostasis and coagulation together with the risk related to warfarin administration are still major concerns in these subjects [13]. Due to the incidence of bleeding complications after LT and their impact on patient survival [14], we preferred a solution bringing the patient to transplantation without anticoagulant treatment, thus minimizing the risk of postoperative hemorrhage. Our choice was supported by the uneventful postoperative course after open heart and both LT procedures and by the excellent long-term result of the aortic valve sparing operation.
